# Automated detection of critical findings in multi-parametric brain MRI using a system of 3D neural networks

**DOI:** 10.1038/s41598-021-86022-7

**Published:** 2021-03-25

**Authors:** Kambiz Nael, Eli Gibson, Chen Yang, Pascal Ceccaldi, Youngjin Yoo, Jyotipriya Das, Amish Doshi, Bogdan Georgescu, Nirmal Janardhanan, Benjamin Odry, Mariappan Nadar, Michael Bush, Thomas J. Re, Stefan Huwer, Sonal Josan, Heinrich von Busch, Heiko Meyer, David Mendelson, Burton P. Drayer, Dorin Comaniciu, Zahi A. Fayad

**Affiliations:** 1 Department of Radiological Sciences, David Geffen School of Medicine at University of California Los Angeles, 757 Westwood Plaza, Suite 1621, Los Angeles, CA 90095-7532 USA; 2 Department of Diagnostic, Molecular and Interventional Radiology, Icahn School of Medicine at Mount Sinai, New York, USA; 3 Digital Technology and Innovation, Siemens Healthineers, Princeton, USA; 4 AI for Clinical Analytics, Covera Health, New York, NY USA; 5 Magnetic Resonance, Siemens Healthineers, New York, USA; 6 Magnetic Resonance, Siemens Healthineers, Erlangen, Germany; 7 Digital Health, Siemens Healthineers, Erlangen, Germany

**Keywords:** Machine learning, Brain imaging, Computer science

## Abstract

With the rapid growth and increasing use of brain MRI, there is an interest in automated image classification to aid human interpretation and improve workflow. We aimed to train a deep convolutional neural network and assess its performance in identifying abnormal brain MRIs and critical intracranial findings including acute infarction, acute hemorrhage and mass effect. A total of 13,215 clinical brain MRI studies were categorized to training (74%), validation (9%), internal testing (8%) and external testing (8%) datasets. Up to eight contrasts were included from each brain MRI and each image volume was reformatted to common resolution to accommodate for differences between scanners. Following reviewing the radiology reports, three neuroradiologists assigned each study to abnormal vs normal, and identified three critical findings including acute infarction, acute hemorrhage, and mass effect. A deep convolutional neural network was constructed by a combination of localization feature extraction (LFE) modules and global classifiers to identify the presence of 4 variables in brain MRIs including abnormal, acute infarction, acute hemorrhage and mass effect. Training, validation and testing sets were randomly defined on a patient basis. Training was performed on 9845 studies using balanced sampling to address class imbalance. Receiver operating characteristic (ROC) analysis was performed. The ROC analysis of our models for 1050 studies within our internal test data showed AUC/sensitivity/specificity of 0.91/83%/86% for normal versus abnormal brain MRI, 0.95/92%/88% for acute infarction, 0.90/89%/81% for acute hemorrhage, and 0.93/93%/85% for mass effect. For 1072 studies within our external test data, it showed AUC/sensitivity/specificity of 0.88/80%/80% for normal versus abnormal brain MRI, 0.97/90%/97% for acute infarction, 0.83/72%/88% for acute hemorrhage, and 0.87/79%/81% for mass effect. Our proposed deep convolutional network can accurately identify abnormal and critical intracranial findings on individual brain MRIs, while addressing the fact that some MR contrasts might not be available in individual studies.

## Introduction

Brain MRI has been established as a powerful and safe diagnostic modality for the majority of neurological disorders by providing detailed evaluation of brain tissue owing to its high soft-tissue contrast. In fact, the Appropriateness Criteria of the American College of Radiology list MR imaging as the desired imaging modality for most neurologic symptoms, including headache, focal neurologic deficits, altered mental status, ataxia, seizure, and vision loss^1^.

A conventional brain MRI study consists of several weightings and is often constructed with at least 5 contrasts including T1-weighted (T1W), T2-weighted (T2W), fluid-attenuated inversion recovery (FLAIR), T2*-weighted (T2*) by using a gradient-recall-echo based sequence, and diffusion-weighted imaging (DWI)^2^. Advances in technology have increased our ability to scan faster, obtaining thousands of high resolution MR images in the order of minutes. Therefore, healthcare providers who own even a few MRI scanners are required to provide interpretation of hundreds of brain MRIs and tens or hundreds of thousands of images on a daily basis. Some of the challenges associated with this increased workload are timely interpretation and triage of scans with acute findings, radiologists’ burnout, and the subjectivity and variability related to the complex nature of modern day large-volume high resolution MR imaging^3^.

One solution to address these challenges is the use of artificial intelligence for automated image classification. It is plausible that an automated and accurate algorithm can provide rapid triage of brain MRIs for critical intracranial findings such as stroke, hemorrhage and mass effect. For large organizations, such triage offers improved radiologist workflow with the potential to reduce the time between acquisition and interpretation for critical cases with presumably positive effects on patient outcome. If the potential of such algorithms is realized, these systems can prioritize radiologic review of cases with critical findings, even if they were not initially ordered for urgent interpretation.

While image interpretation and classification has been a target of computer-aided diagnosis and machine learning techniques for quite some time^4–9^, algorithms have been limited by data set size constraints and reliance on experts’ feature selection, resulting in limited ability to handle image diversity and complexity. In recent years, increased access to large data and computational capabilities have enabled deep-learning (DL)-based algorithms in the field of medical imaging that are capable of learning complex patterns from large image data sets at multiple levels of abstraction, representation and information, without the need for predefined feature extraction, and with promising performance and speed^10–14^. Although progress has been made in automated triage of non-contrast head CT^15,16^, applications for triaging multiple critical findings on brain MRI are sparse.

The contributions of this paper lie in applying DL concepts to automate the classification of patients’ brain MRI studies. Specifically, we developed and measured the performance of the first DL-based prototype to both differentiate between normal brain and abnormal brain MRI studies and identify critical intracranial findings including acute infarction, acute hemorrhage and mass effect using brain MRI. The proposed methodology uses a custom DCNN architecture that accommodates variable sets of image contrasts and combines localization feature extraction (LFE) modules with a global classifier that handles images from multiple orientations.

## Methods

### Data

This study was compliant with the health insurance portability and accountability act (HIPAA). Mount Sinai institutional review board approved this study for human research with waiver of informed consent. All methods were performed in accordance with the relevant guidelines and regulations.

**Figure 1:**
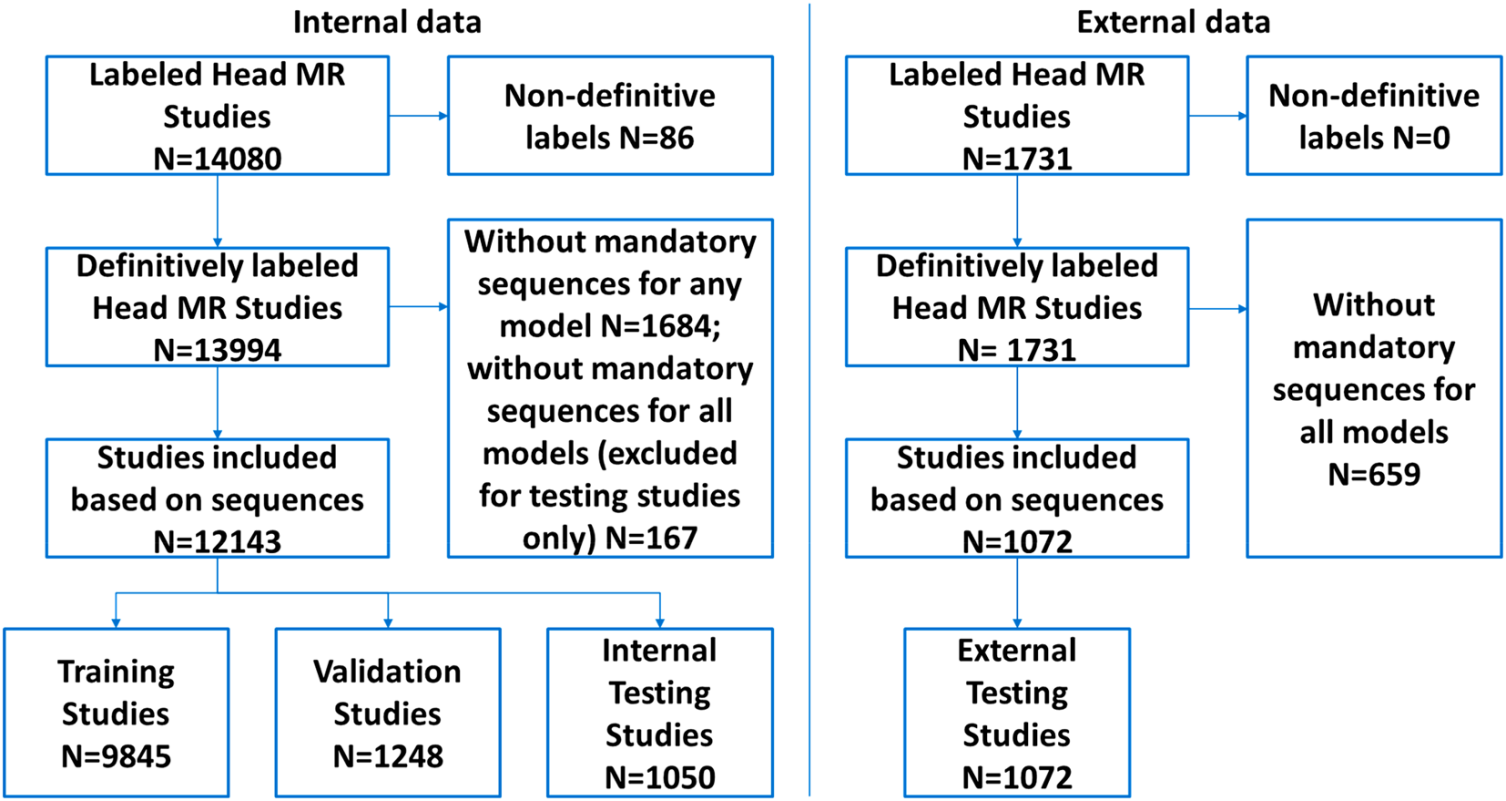
Study flowchart. Non-definitive labels denote reports where the neuroradiologists deemed that classifying the study as positive or negative for one or more of the labels would be acceptable.

A total of 15,811 brain MRI studies were used in this study: an internal data set of 14,080 studies used for training and internal testing, and an external data set of 1731 studies used for external testing. The internal data set was accumulated from a combination of inpatient and outpatient scanners from our institution’s HIPAA compliant imaging research warehouse, including data from 19 scanners produced by two manufacturers (GE and Siemens Healthcare). The external data was accumulated from two external centers, including data from scanners produced by three manufacturers (GE, Philips, and Siemens Healthcare). Studies were filtered based on MR contrasts available, spatial characteristics and label ambiguity (inclusion/exclusion flowchart in Fig. 1; filter details in Supplementary materials), yielding 12,143 internal studies that were randomly assigned to training (74%), validation (9%) and internal testing (8%) sets using a single patient-wise split of data from all scanners and 1072 external studies for further testing (8%).

Each of the studies was labeled based on a re-analysis of each radiology report by one of two board-certified neuroradiologists (AD and KN with at least 10 years of experience each) for internal studies and by a third radiologist (TR) for the external studies to denote the following:

- presence of any intracranial abnormality (any intracranial findings even minor or of doubtful clinical significance were considered as abnormal, i.e. any white matter signal change, developmental venous anomaly, pineal cyst, …). Therefore, a normal brain MRI was considered as one with absolutely no parenchymal signal change and no incidental findings.
- acute infarction: any areas of infarction reported as having reduced diffusion were included. Chronic infarction description was excluded from this label class.
- acute hemorrhage: subarachnoid, intraparenchymal or extra-axial hemorrhages were included. Any description of chronic hemorrhage was excluded from this label class.
- mass effect: mass effect worthy of notification with any description of mid-line shift, effacement of basal or suprasellar cisterns, or uncal herniation was included. Localized mass effect surrounding a mass without causing midline shift or effacement of sulci was not included in this category.

To assess the interobserver variability in labeling the critical findings from radiology reports, a set of reports from 200 patients were reviewed independently by both neuroradiologists.

The 3D boundaries of critical findings were delineated by a radiologist (TR). For acute infarction, reduced diffusion was delineated on TraceW images, defined by its hyperintense signal. For acute hemorrhage, hemorrhage excluding surrounding edema was delineated on FLAIR images, using both hyperintensity on FLAIR and hypointensity on T2* images to assess hemorrhage extent. For mass effect, the regions of tissue that were visibly displaced were delineated on FLAIR images, excluding any mass causing the displacement.

### Image analysis

Images were preprocessed by resampling them to the coordinate grid of a reference contrast (axial ADC and sagittal T1w for abnormality, axial FLAIR for hemorrhage and mass effect models, and axial ADC for the infarct model) based on scanner coordinates, and resizing them to a fixed dimension [256 × 256 in-plane and 32 out-of-plane]. Image intensities of each contrast were normalized using a linear transformation of minimum and maximum values to 0–1).

#### Network architecture

**Figure 2:**
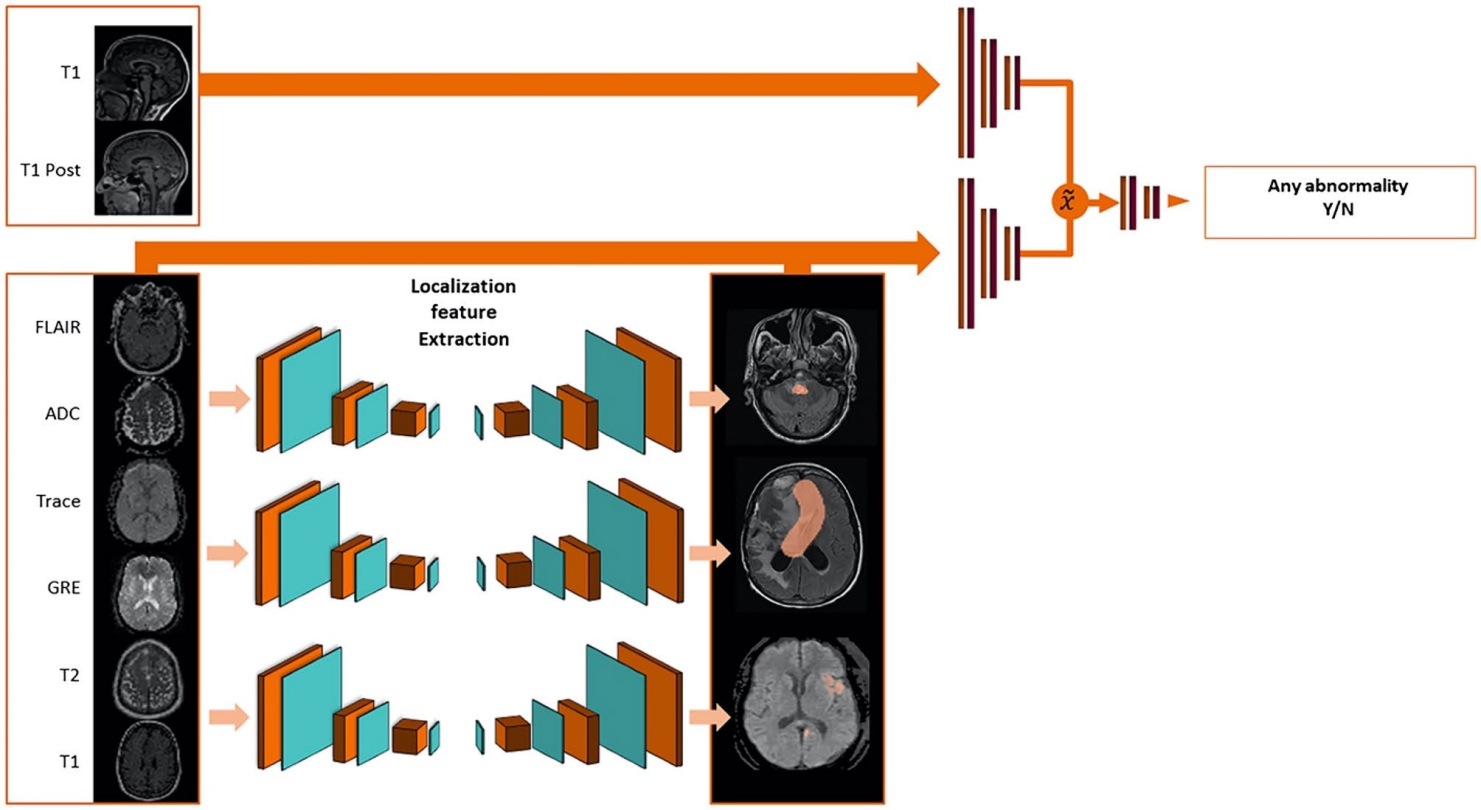
Abnormality network architecture.

**Figure 3:**
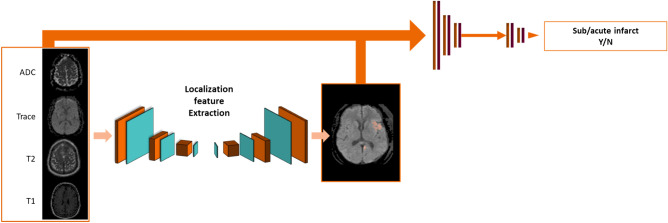
Critical network architecture. The same architecture is used for acute hemorrhage, mass effect and acute infarct, but is trained using different inputs. The network for infarct is shown above; the corresponding network for mass effect used axial FLAIR, T1W and T2W contrasts, and the network for hemorrhage used all axial contrasts. Note that for all three critical findings, the input contrast selection (see ‘training’ section) resulted in sagittal images not being used, simplifying the architecture compared to Fig. 2.

The classification of each study is performed by 4 networks: one to detect the presence of any abnormality and three to detect the three acute abnormalities. Each network was trained on a network-specific set of images (detailed in Table 1). The overall architectures for the abnormality and critical finding networks are shown in Figs. 2 and 3, respectively and summarized below. Architecture implementation details and training hardware are detailed in the Supplementary materials.

**Table 1:** Contrasts used for each classification.

Contrast	Any abnormality	Hemorrhage	Infarct	Mass effect
Axial T2 FLAIR	Required	Required		Required
Axial ADC	Required	Required	Required	
Axial Trace-weighted	Required	Required	Required	
Axial T2*-weighted	Optional	Optional		
Axial T1-weighted	Optional	Optional	Optional	Optional
Sagittal T1-weighted	Optional			
Sagittal T1-weighted post-contrast	Optional			
Axial T2-weighted	Optional	Optional	Optional	Optional

The network comprises three components: (1) localization feature extraction (LFE) modules that create a pixel-wise map of the findings on the axially oriented volumes; (2) orientation-specific feature-combination layers and (3) a global classifier that generates a study-wise score for the presence of the finding.

The localization feature extraction modules are deep image-to-image networks^17^ comprised of a series of convolution and pooling layers generating successively lower resolution feature maps, followed by a series of convolution and up-sampling layers successively restoring the original resolution, with skip connections connecting the two sections to transmit higher resolution information.

The orientation-specific feature combination layer is designed to make our algorithm compatible with different clinical protocols that may vary between sites. For the detection of any abnormality, for example, only FLAIR, ADC and TraceW are required contrasts, while the others are considered as optional. The orientation-specific feature combination layer uses a separate in-plane convolution for each contrast present, and then averages the resulting features within each orientation, excluding those from missing contrasts. This averaging makes the network robust to distributional shifts caused by missing contrasts, and induces the features from different contrasts to encode information in a common space.

Then, the classifier uses a series of in-plane strided convolutions for each orientation to yield isotropic outputs. In the abnormality network, which uses both sagittal and axial images (see Table 1), the orientation-specific outputs are averaged when sagittal images are present. The resulting outputs are inputted into a final series of convolution and pooling layers. This is followed by a global pooling layer with softmax to score a finding from 0 to 1 as present or absent. The operating point of this classifier can be tuned to prioritize fewer false positives or fewer false negatives; for the analysis reported in this paper, the operating point was tuned on the validation set to minimize the sum of sensitivity and specificity.

#### Training

The networks were trained in two stages. First, the LFE networks were trained in isolation; then, the full network was trained end-to-end to generate a study-wise prediction. The LFE networks were trained minimizing the pixel-wise cross-entropy loss compared to manual annotations of the critical findings. The classifier networks were trained minimizing the cross-entropy loss compared to the study-wise manual label. Both types of networks were trained with an Adam-type optimizer^18^ for up to 500 epochs with early stopping based on the loss on the validation set, actually stopping after 7–42 epochs. The training algorithm used balanced positive/negative data sampling to mitigate class imbalance, and the data was augmented using randomized spatial transformations (translation and left–right flipping) and intensity transformations (additive uniform noise^19^) to mitigate overfitting. Dropout was used in each convolution layer to mitigate overfitting.

The network architecture, hyperparameters and input contrasts were iteratively optimized based on the ROC-AUC on the validation set using the single original split. The validation set was also used to set the operating point for the classifiers as the threshold that maximizes the sum of sensitivity and specificity.

#### Network interpretability

**Figure 4:**
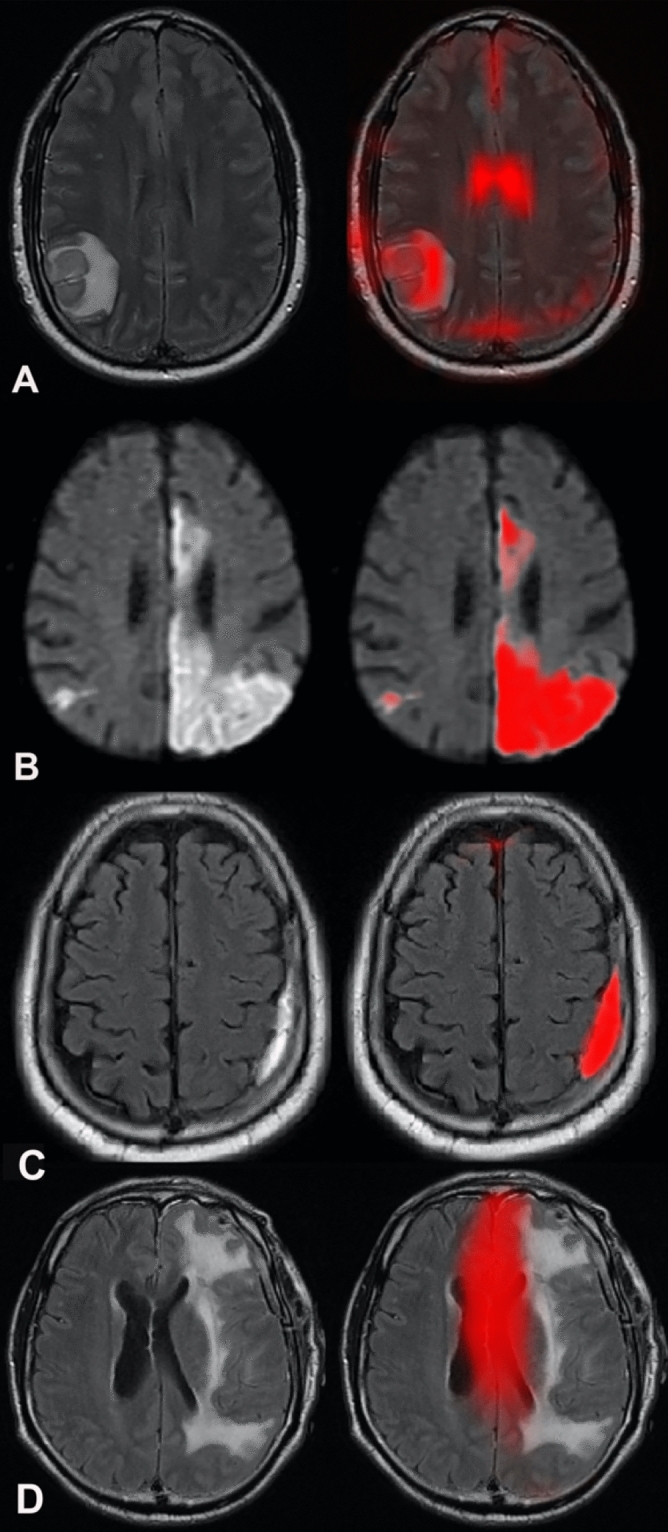
Network interpretability. For the abnormality classification, a heat map can be generated retrospectively denoting regions that strongly influenced the decision: (**A**). For critical findings, the network directly generates heat maps of denoting the region identified by the AI: acute infarction on a trace-weighted contrast (**B**), subdural hemorrhage on a FLAIR contrast (**C**) and mass effect on a FLAIR contrast (**D**).

To give insight into the network’s predictions, heat maps are generated for each contrast to indicate the importance of the input pixel. For the critical finding models, the localization feature extraction module generates this directly. For the abnormality model, the importance is computed in latent feature space by taking a gradient of the network’s output signal, and then this is scaled to each input contrast dimension to provide the pixel-wise importance in input space^20^. Examples of network output are shown in Fig. 4.

### Statistical analysis

Identification on brain MRI of any abnormality, acute hemorrhage, acute infarction and mass effect on a per-study basis were considered as primary end points of this study. A given MRI study was considered positive for a finding if the classification score exceeded the corresponding operating point. Receiver operative characteristic analysis was performed to calculate the network performance including the area under the curve, sensitivity, specificity, negative and positive predictive value and diagnostic accuracy in both internal and external test groups. Interobserver agreement for labeling the critical findings was measured by using Cohen’s kappa score.

## Result

From a total of 14,080 brain MRI studies, 12,143 met our inclusion criteria (Fig. 1). Among these studies, 10,140 (84%) were abnormal, 1219 (10%) had acute infarction, 533 (4.4%) had acute hemorrhage and 550 (4.5%) had intracranial mass effect. There were 404 studies with multiple findings (59 with mass effect and infarct, 195 with hemorrhage and infarct, 88 with hemorrhage and mass effect, and 62 with all three); as the findings were classified independently, these cases were treated as positive for each of the respective findings.

Interobserver agreement for labeling critical findings was excellent for acute infarct (k = 0.93); excellent for acute hemorrhage (k = 0.84); and very good for mass effect (k = 0.77).

Among the internal testing group (1050 brain MRIs), 879 (84%) were abnormal, 118 (11%) had acute infarction, 65 (6.2%) had acute hemorrhage, and 56 (5.3%) had intracranial mass effect. Among the external testing group (1072 brain MRIs), 867 (81%) were abnormal, 287 (27%) had acute infarction, 78 (7.3%) had acute hemorrhage and 31 (2.9%) had intracranial mass effect.

The diagnostic performance of our deep neural network for the internal and external test sets are summarized in Tables 2 and 3, respectively.

**Table 2:** Performances of the classifiers on the internal test set.

Detection type	AUC	Sensitivity (%)	Specificity (%)	ACC (%)	PPV (%)*	NPV (%)*
Abnormality	0.91	83	86	84	97	50
Acute hemorrhage	0.90	89	81	81	24	99
Acute infarct	0.95	92	88	88	49	99
Mass effect	0.93	93	85	85	26	100

*Note that positive predictive value (PPV) and negative predictive value (NPV) are highly dependent on the prevalence of the finding in a given population.

**Table 3:** Performances of the classifiers on the external test set.

Detection type	AUC	Sensitivity (%)	Specificity (%)	ACC (%)	PPV (%)*	NPV (%)*
Abnormality	0.88	80	80	80	94	48
Acute hemorrhage	0.83	72	88	87	32	98
Acute infarct	0.97	90	97	95	92	96
Mass effect	0.87	79	81	81	12	99

*Note that positive predictive value (PPV) and negative predictive value (NPV) are highly dependent on the prevalence of the finding in a given population.

The full spectrum and amplitude of acute findings were not available through the radiology reports for consistent labeling and testing via our classifiers. However, for the size of infarction, we were able to perform a sub-analysis between the impact of infarct volume (obtained from manual contours in the testing group) and the performance of our model. This analysis did show a downward trend in diagnostic performance with the smaller size of infarction with calculated sensitivity of 84% for infarction volume < 1 mL, 79% for infarction volume < 0.5 mL, and 72% for infarction volume < 0.25 mL.

**Figure 5:**
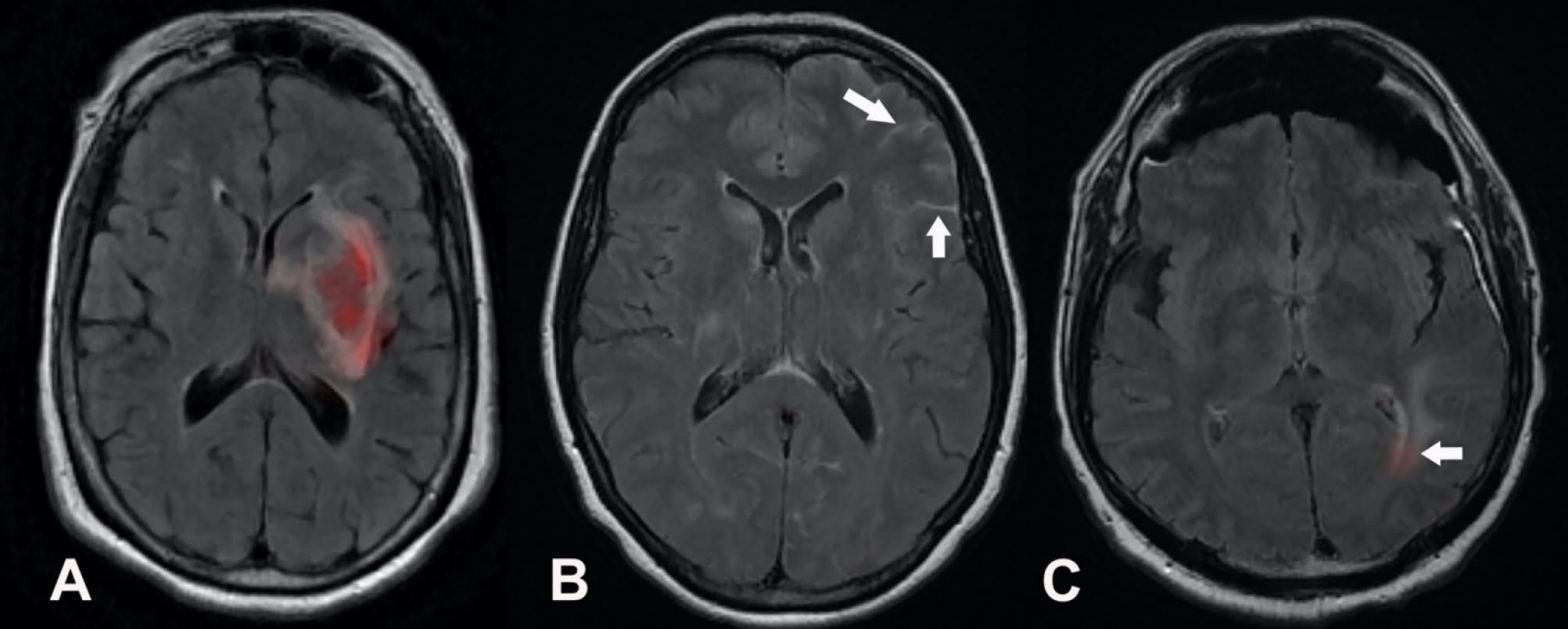
Examples of heat maps provided by the network for detection of acute intracranial hemorrhage. (**A**) Heatmap on axial FLAIR shows correct identification of intraparenchymal hemorrhage (true positive). (**B**) Subarachnoid hemorrhage seen as sulcal hyperintense signal on axial FLAIR image (arrows) was not detected by the network (false negative). (**C**) Heatmap on axial FLAIR points to periventricular leukomalacia (arrow) (false positive).

Figure 5 shows examples of true positive, false negative and false positive in acute hemorrhage detection by our network.

To assess the contributions of the optional contrasts, we additionally measured the performance of the models when given only the mandatory sequences. The change in performance of these models are summarized in Table 4. Overall, with only the mandatory contrasts (chosen for their clinical value) the models achieved performance close to that with all available contrast, with the difference much less pronounced for the external data set. Most notably, the inclusion of T1w, T2w and T2* made the hemorrhage model substantially more specific, though less sensitive.

**Table 4:** Contribution of optional contrasts to classifier performance.

Data set	Detection type	Δ AUC	Δ Sensitivity (%)	Δ Specificity (%)
Internal	Abnormality	0.01	2	1
	Acute hemorrhage	0.01	− 2	6
	Acute infarct	0.00	0	0
	Mass effect	0.01	3	1
External	Abnormality	0.00	0	0
	Acute hemorrhage	0.01	− 2	3
	Acute infarct	0.00	0	0
	Mass effect	0.01	2	− 2

Positive values denote that the model performance was higher with all available contrasts than with only mandatory contrasts.

## Discussion

Our results showed that the proposed automated DCNN-based tool can identify normal versus abnormal brain MRIs and flag critical findings including acute infarction, hemorrhage and mass effect with acceptable diagnostic accuracy on routine brain MRI scans from multivendor imaging systems.

In this work, we designed a DCNN architecture that integrates global classification of pathology with automatic localization feature extraction in the form of pixel-level maps denoting the locations of critical findings. This architecture enables the training process to inject additional complex clinical understanding of the MR images through deep supervision with clinical contours. Through a later combination of these maps with the original images, they also act as an attention mechanism on the relevant image information that determines the final decision that is not only used by the classification, but also can be visualized to understand the workings of the network.

Although DL methods have been used to identify multiple critical intracranial findings on CT^21,22^, available solutions to triage multiple acute intracranial findings on brain MRIs have been relatively limited to a few applications^23,24^. Below we discuss our specific results as they relate to acute infarction, acute hemorrhage, and mass effect.

Timely diagnosis of acute stroke is crucial, since reducing the time to treatment by 15 min has resulted in significant improvement in final outcome in patients with acute stroke^25^. It is therefore critical for institutions to rapidly and accurately interpret neuroimaging data in the presence of acute ischemic stroke. While MRI (DWI) is the most accurate imaging modality for detection of acute infarction^26^, CT has been the major workhorse for acute stroke imaging due to its broad availability. Therefore, there has been a paucity of data on prior work for automated detection of acute stroke on MRI using DL techniques. Though not intended for detection of acute infarction, Chen et al.^27^ used a framework with two CNNs to segment stroke lesions in known stroke patients using DWI with a reported mean Dice coefficient of 0.67.

Intracranial hemorrhage (ICH) is another critical medical event with reported mortality of up to 40%^28^ which can occur both within the brain parenchyma (intra-axial) or external to the brain parenchyma (extra-axial including epidural, subdural and subarachnoid). Although automated detection of ICH has been investigated with deep learning algorithms on CT with promising results^15,16^, automated detection of ICH on MRI has been limited to small series for detection of parenchymal microbleeds^29^. To our knowledge, no prior studies looked at automated detection of all categories of acute ICH on MRI via deep learning. Our DL algorithm was able to detect acute ICH successfully with an AUC of 0.90 comparable to reported results of DL algorithms applied on CT with AUCs of 0.91^21^ and 0.94^22^.

Intracranial mass effect is also a serious condition that can cause brain shift, followed by herniation, brainstem compression, and death. In the NIH Traumatic Coma Data Bank, midline shift and compression or obliteration of the perimesencephalic cisterns were among the most important imaging characteristics associated with poor outcome and death^30^. Early recognition can prompt therapeutic interventions and improve outcome. Recently, DL methods have been applied for detection of intracranial mass effects on head CT with reported AUCs in the range of 0.86–0.91^21,22^. Our DL approach was able to identify intracranial mass effect with an AUC of 0.93 and is the first of its kind for detection of mass effect using MRI.

In testing our single-center model on external data from multiple centers, we observed a shift in performance but no consistent trend across the models. For mass effect, the sensitivity and specificity each decreased by ~ 10%, while for acute infarct, the specificity increased by the same margin with little change in sensitivity. For acute hemorrhage, there was a shift in operating point to lower sensitivity and higher specificity. This suggests two strategies might be applied for developing such systems: training multi-center models for more stable multi-center generalization, or training single-center models optimized for the characteristics of a given center.

Accurate and timely diagnosis of critical intracranial findings such as stroke, ICH and mass effect is crucial for proper management. Increased use of neuroimaging in large clinical practices and interruptions from noninterpretive tasks common in academia can result in delayed diagnosis of these critical findings. The turn-around time for simple non-contrast head CT has been reported in the range of 1.5–4 h in the emergency departments^31^. The turn-around time for outpatient non-urgent studies have been set and accepted within 24 h across many institutions such as ours due to shortage of human resources. These delays however can impact patients’ care and negatively impact the patient’s outcome.

An automated screening tool such as our DL algorithm may therefore have the potential for efficient and accurate diagnosis of acute intracranial findings to facilitate a prompt therapeutic response. The most salient use case of our algorithm is a screening system that can alert physicians about MRI examinations with positive critical findings for expedited interpretation and reduced turn-around time, which has been rated as one of the highest priorities in a recent survey of > 80 imaging institutions^32^.

Several variables can determine the urgency of any given study in radiology workflow. Some of these are (1) location of ordering physician (Emergency department, inpatient, outpatient clinic); (2) priority level assigned by ordering physicians (high priority, routine); and (3) information obtained based on patients symptoms (stroke, trauma, intracranial hemorrhage). Although this style of prioritization serves as a guide to determine the urgency and order by which the studies can be interpreted, AI algorithm such as ours can provide a different insight and reprioritize the list based on actual findings contained in the images for more realistic and less assuming model. For example, a patient with headache and confusion may be assigned a routine brain MRI with potential waiting time-to-interpretation of several hours. However, if this patient has a stroke, using our classifier this study will be prioritized despite having a routine order and will be read at the top of the worklist by next available radiologist. Although this process was not tested systematically in our work (due to retrospective design), our results support the potential of such an algorithm/model.

Another potential use case for our classifiers is for institutions with multiple imaging centers such as ours that function across a large urban area and well beyond routine working hours. We envision that in such practice setting, the classifiers described in our work can be in place to screen and flag MRI scans with critical intracranial findings as they are being obtained so a notification can be sent to a central reading rooms that function 24/7 for timely interpretation of these studies rather than waiting until the next morning for day-time radiologist.

Depending on the prevalence of acute findings and clinical preference in a given practice, the operating point of the system can be tuned, without retraining, to optimize for different clinical priorities, such as prioritizing sensitivity to catch all serious findings. The potential benefits of this system are to improve work-flow during working hours to tailor available human expertise in interpreting abnormal cases in a timely manner, and identify patients with critical intracranial findings during off hours to notify emergency and on-call personnel, so no urgent MRI studies will go untriaged.

One of the advantages of the model described in this work is that the combination of FLAIR and diffusion imaging is an integral part of all of our modules. This supports the broadly accepted concept of having diffusion MRI as the most accurate diagnostic modality for acute infarction^26^ and FLAIR as the most versatile contrast for detection of the majority of all intracranial pathologies^33^. Having a system that can classify MR images with only these contrasts or with additional ones provides much needed flexibility for extending our model to data sets generated at most neuroimaging institutions, as these contrasts are almost universally included in all brain MRI studies.

Our study has several limitations. First, its retrospective nature introduces unknown bias. Second, despite our promising results that was tested both internally and on an external test group, a larger validation study is required. Finally, the study was limited to analyzing the performance of the system in isolation. While fully-automatic image-based classification has the potential to improve patient outcomes and radiology workflow through improving the triage of acute findings and treatment, it is the authors’ collective opinion that such an algorithm can ultimately be developed to provide decision support combining imaging data, clinical data and interactive input from physicians and radiologists.

## Conclusion

Our proposed deep learning 3D neural network can accurately identify critical findings on individual brain MRIs from a flexible selection of MR contrasts. If its potential is realized it can be used to flag abnormal brain MRIs, improving triage and timely interpretation of abnormal scans in a busy clinical practice.

## Supplementary Information

Supplementary Information 1

Supplementary Information 2

Supplementary Information 3.

Supplementary Information 4

Supplementary Information 5

Supplementary Information 6

Supplementary Information 7

Supplementary Information 8

Supplementary Information 9

## Data Availability

The datasets used in this study are not publicly available because the IRB of the study limits access to the data. Derived and supporting data are available from the corresponding author upon reasonable request. We are willing to validate other people's models as part of collaborations. The code used for training the models has a large number of dependencies on internal tooling, infrastructure and hardware, and its release is therefore not feasible. However, an independent library to construct the network architectures and to load data as used in this study have been made available to support replication with non-proprietary libraries.
