# Supplementary material for: Automated detection of critical findings in multi-parametric brain MRI using a system of 3D neural networks
Source: Sci Rep. 2021 Mar 25;11:6876. doi: 10.1038/s41598-021-86022-7 (PMC7994311; doi:10.1038/s41598-021-86022-7)
Supplement: Supplementary file 1 — Supplementary Information 1 [file 41598_2021_86022_MOESM1_ESM.docx]

**Automated Detection of Critical Findings in Multi-Parametric Brain MRI using a system of 3D neural networks**

Kambiz Nael^1,2*^, MD; Eli Gibson, PhD^3^; Chen Yang, PhD^2^; Pascal Ceccaldi, MSc^3^; Youngjin Yoo, PhD^3^; Jyotipriya Das, MSc^3^; Amish Doshi, MD^2^; Bogdan Georgescu, PhD^3^; Nirmal Janardhanan^3^; Benjamin Odry, PhD^4^; Mariappan Nadar, PhD^3^; Michael Bush, PhD^5^; Thomas J. Re, MD^3^; Stefan Huwer^6^; Sonal Josan, PhD^3^; Heinrich von Busch, PhD^3^; Heiko Meyer, PhD^6^; David Mendelson, MD^2^; Burton P. Drayer, MD^2^; Dorin Comaniciu, PhD^3^; Zahi A. Fayad, PhD^2^

**Supplementary materials:**

**a) Data filtering**

The studies were filtered to select those that met the following criteria:

1. Input data should adhere to the anatomical orientations described in Table 1 of the main manuscript.

2. Patient age should be ≥ 18 years

3. Image Orientation Patient (DICOM tag (0020,0037)) should be same across slices for each contrast

4. Pixel Spacing (DICOM tag (0028,0030)) should be same across slices for each contrast

5. Slice thickness (DICOM tag (0018,0050)) should be same across slices for each contrast

6. Slice distance should be same across slices for each contrast

7. Magnetic field strength (DICOM tag (0018,0087)) should be 1·5 or 3 Tesla

8. TraceW image b-value should be 1000 s/mm^2^

9. Input data should be in the recommended resolution ranges mentioned in **Appendix Table 1.**

**Appendix Table 1: Spatial characteristics and resolutions used in data filtering.**

|  | | | | | | | | | | | | | | | | |
| --- | --- | --- | --- | --- | --- | --- | --- | --- | --- | --- | --- | --- | --- | --- | --- | --- |
|  |  | | |  | |  | | |  | | | |  | |  | |
| **Contrast Type** | **Pixel spacing  (in-plane, mm)** | | **Slice thickness**  (mm) | | | | **Spacing between slices** (mm) | | | **Slice Distance (mm)** | | **Field of View  (out-of-plane, mm)** | | | | **# slices** |
|  | Min | Max | Min | | Max | | Min | Max | | Min | Max | Min | | Max | | Min |
| Sagittal T1-weighted | 0·2 | 1·1 | 0·5 | | 5 | | 0·5 | 8 | | 0·5 | 8 | 100 | | 400 | | 15 |
| Sagittal T1-weighted post-contrast | 0·2 | 1·1 | 0·5 | | 5 | | 0·5 | 8 | | 0·5 | 8 | 100 | | 400 | | 15 |
| Axial T1-weighted | 0·2 | 1 | 0·5 | | 5 | | 0·5 | 8 | | 0·5 | 8 | 100 | | 400 | | 20 |
| Axial T2-weighted | 0·2 | 1·1 | 0·5 | | 5 | | 0·5 | 8 | | 0·5 | 8 | 100 | | 400 | | 20 |
| Axial ADC | 0·3 | 1·9 | 0·9 | | 5 | | 0·9 | 8 | | 0·9 | 8 | 100 | | 400 | | 20 |
| Axial Trace-weighted | 0·3 | 1·9 | 0·9 | | 5 | | 0·9 | 8 | | 0·9 | 8 | 100 | | 400 | | 20 |
| Axial T2 FLAIR | 0·2 | 1 | 0·5 | | 5 | | 0·5 | 8 | | 0·5 | 8 | 100 | | 400 | | 20 |
| Axial T2*-weighted | 0·2 | 1·2 | 0·5 | | 7 | | 0·5 | 8 | | 0·5 | 8 | 100 | | 400 | | 15 |

**b) Network architecture**

The general architecture of the networks is shown in Figure 3 and 4. For reproducibility, the networks can be constructed in pytorch using network.AbnormalityNetwork, network.HemorrhageNetwork, network.InfarctNetwork, and network.MassEffectNetwork in the included python code. The abnormality network is based on networks.FullNet network which uses both sagittal and axial images, and the critical finding classifiers are based on networks.AxialNet which use only axial images.

We include a brief description of the networks here. Unless otherwise specified, convolution layers refer to 3D convolutions with instance normalization, 20% dropout, and ReLU activation, while pooling layers refer to 3D max pooling with stride 2.

The LFE networks, encoded as masked_unet.MaskedUNet in the included python code, used 4 sets of downsampling convolution/pooling layers followed by 4 sets of upsampling convolution layers, with skip connections. The downsampling section used 16 initial channels with an additional 16 channels after each pooling. The first of these convolutions averages the output channels from each input contrast that is available instead of summing them, for robustness to missing contrasts. The upsampling section used nearest-neighbor interpolation and convolutions with 16 channels. Skip connections were introduced by concatenation. This was followed by a final convolution with 2 channels and no activation as the final features. In this final layer, the normalization of the features differed between models, with the mass effect model using instance normalization, and the others using no normalization. The critical finding models each use one LFE network for the respective finding, whereas the abnormality model uses three LFE networks, one for each finding and concatenates the resulting features.

The classification modules have two sections: an orientation-specific section for each orientation that encodes the LFE outputs and original contrasts to an isotropic set of computed features, and a common classification section that uses the isotropic set of features to compute the final prediction. The orientation-specific section, encoded as orientation_specific.OrientationSpecific in the included python code, consists of 3 anisotropic convolutions with kernel size 3 and stride 2 in-plane and kernel size 1 and stride 1 out-of-plane. The first of these convolutions averages the output channels from each input contrast that is available instead of summing them, for robustness to missing contrasts. The common classification section, encoded as fcn.FCN in the included python code, consists of 4 sets of convolution/pooling layers with 16, 32, 64 and 2 channels with the last pooling layer being a global max pooling layer.

**c) Optimizer hyperparameters**

The networks were trained with separate optimizer hyper-parameters which can be constructed using the included python code, as optimizers.AbnormalityOptimizer, optimizers.HemorrhageOptimizer, optimizers.InfarctOptimizer, optimizers.MassEffectOptimizer, differing in the use of Adam (for hemorrhage) or AdamW (for the others), and in the learning rate and weight decay parameters, as selected during hyperparameter selection.

**d) Data preprocessing**

The data loading and preprocessing is implemented as data.load_study in the included python code.

**e) Training software and hardware**

Image preprocessing was performed using python. Networks were implemented in pytorch v1.3.0 using CUDA v10.1 and CUDNN v7.0. Experiments were performed on an Intel Xeon Gold 6148 40-core server with 8 NVIDIA Tesla V100 SXM2 16GB GPUs. The training time for the models included 8-30 hours for training the localization feature extraction modules and 45-98 hours for training the full system. After training the system, the processing time for prediction in pytorch for a single MR study is 25 seconds using a GPU and 33 seconds without.
